# The Essential Role of *Drosophila* HIRA for De Novo Assembly of Paternal Chromatin at Fertilization

**DOI:** 10.1371/journal.pgen.0030182

**Published:** 2007-10-26

**Authors:** Emilie Bonnefoy, Guillermo A Orsi, Pierre Couble, Benjamin Loppin

**Affiliations:** 1 Université Lyon 1, Lyon, France; 2 CNRS, UMR5534, Centre de Génétique Moléculaire et Cellulaire, Villeurbanne, France; European Molecular Biology Laboratory, Germany

## Abstract

In many animal species, the sperm DNA is packaged with male germ line–specific chromosomal proteins, including protamines. At fertilization, these non-histone proteins are removed from the decondensing sperm nucleus and replaced with maternally provided histones to form the DNA replication competent male pronucleus. By studying a point mutant allele of the *Drosophila Hira* gene, we previously showed that HIRA, a conserved replication-independent chromatin assembly factor, was essential for the assembly of paternal chromatin at fertilization. HIRA permits the specific assembly of nucleosomes containing the histone H3.3 variant on the decondensing male pronucleus. We report here the analysis of a new mutant allele of *Drosophila Hira* that was generated by homologous recombination. Surprisingly, phenotypic analysis of this loss of function allele revealed that the only essential function of HIRA is the assembly of paternal chromatin during male pronucleus formation. This HIRA-dependent assembly of H3.3 nucleosomes on paternal DNA does not require the histone chaperone ASF1. Moreover, analysis of this mutant established that protamines are correctly removed at fertilization in the absence of HIRA, thus demonstrating that protamine removal and histone deposition are two functionally distinct processes. Finally, we showed that H3.3 deposition is apparently not affected in *Hira* mutant embryos and adults, suggesting that different chromatin assembly machineries could deposit this histone variant.

## Author Summary

Chromatin is composed of basic units called nucleosomes, in which DNA wraps around a core of histone proteins. HIRA is a histone chaperone that is specifically involved in the assembly of nucleosomes containing H3.3, a universally conserved type of histone 3. To understand the function of HIRA in vivo, the authors generated mutant fruit flies with a non-functional Hira gene. Surprisingly, mutant flies were viable, but females were completely sterile. By analysing the female fruit flies' eggs, the authors found that in the absence of HIRA protein, the sperm nucleus was unable to participate in the formation of the zygote. In *Drosophila,* as in many animals, the condensed sperm chromatin contains protamines instead of histones. The authors found that the only crucial role of HIRA in flies was to assemble nucleosomes containing H3.3 in the male pronucleus, after the removal of protamines. This fundamental process, which is presumably also controlled by HIRA in vertebrates, allows the paternal DNA to reconstitute its chromatin and participate in the development of the embryo.

## Introduction

The assembly of nucleosome particles on nuclear DNA is the initial step for the formation of chromatin. Nucleosome assembly initiates with the formation of a H3-H4 histone tetramer on DNA followed by the addition of two H2A-H2B dimers to form the octameric particle [1,2]. Although this organisation of genomic DNA is remarkably conserved in eukaryotes, sperm cells of many species are characterized by a very different type of chromatin architecture involving non-histone proteins such as protamines [3]. The replacement of histones with protamines or other sperm nuclear basic proteins (SNBPs) during the differentiation of post-meiotic spermatids is generally associated with a high level of nuclear condensation, a general shutdown of transcriptional activity, and a state of chromatin that is incompatible with DNA replication [3–5]. Although the precise advantages of acquiring a specialized type of chromatin for the sperm cell are poorly known, the protamine type of chromatin could protect the paternal DNA from damaging agents or allow the resetting of epigenetic marks carried by histones [6–8]. In any case, once entered in the egg cytoplasm, the fertilizing sperm nucleus must replace its SNBPs with maternally provided histones that are stored in the egg cytoplasm. This process, called sperm chromatin remodelling (SCR), allows the paternal DNA to recover a nucleosomal chromatin and thus guarantees the ability of the male pronucleus to replicate its DNA in coordination with its female counterpart [3–5]. SCR can be separated into two key processes. The first process is the removal of SNBPs from the paternal DNA once the sperm nucleus is released in the egg cytoplasm. The second is the assembly of nucleosomes from maternal components before the first round of DNA replication. SCR has been almost exclusively studied in animal models that produce large quantities of eggs, such as amphibians or sea urchins, thereby facilitating the biochemical characterization of factors capable of remodelling sperm nuclei in vitro [3]. *Drosophila* embryonic extracts have also been used as a source of sperm chromatin decondensation factors [9–12], but none of the identified molecules has been demonstrated so far to have a function in SCR in vivo. In *Drosophila*, the sperm DNA is packaged with two protamines, whereas core histones are not detectable in male gamete nuclei [13,14]. In this sense, *Drosophila* represents a good model for the functional study of SCR in vivo. In previous publications, we characterized *sésame (ssm)*, a *Drosophila* maternal effect mutation that specifically prevented male pronucleus formation [15] and SCR [16]. This mutation was subsequently shown to cause a single amino acid substitution (R225K) in the *Hira* gene [17].

HIRA is a conserved chromatin assembly factor that allows the replication-independent (RI) deposition of core histones on DNA, in contrast to the CAF-1 complex whose replication-coupled (RC) nucleosome assembly activity is strictly linked to DNA synthesis [18]. Accordingly, it has been established in vitro that HIRA specifically deposits H3-H4 dimers that contain the histone H3 variant H3.3, which is expressed throughout the cell cycle, whereas CAF-1 deposits H3-H4 dimers that contain the replicative histone H3.1 [19]. Our functional analysis of the *Drosophila Hira* gene allowed us to demonstrate in vivo that HIRA was indeed involved in the RI deposition of H3.3 [17]. In addition, we observed that maternal HIRA localized in the decondensing sperm nucleus where it deposited H3.3-H4 histones before the first zygotic S phase, thus establishing the essential role of HIRA in SCR. Recently, the *Hira^ssm^* allele was found to enhance the variegation of a *white* reporter transgene, indicating that HIRA could help counteract the spread of heterochromatin by mediating histone replacement at specific sites [20]. However, because of the subtle nature of the *Hira^ssm^* mutation and the absence of obvious phenotype in mutant adults, it was not clear whether HIRA could have important functions during development or in adult flies. In this paper, we report the characterization of a loss of function *Hira* allele that we have generated by homologous recombination. Surprisingly, we show that paternal chromatin assembly at fertilization is the only developmental process that absolutely requires HIRA. We also demonstrate that protamine removal does not depend on HIRA and is thus functionally distinct from the paternal nucleosome assembly process. Finally, we show that H3.3 is deposited in the chromatin of mutant embryos and adults, suggesting that other factors are implicated in the assembly of H3.3 nucleosomes.

## Results

### Targeting the *Hira* Gene by Homologous Recombination

**Figure 1:**
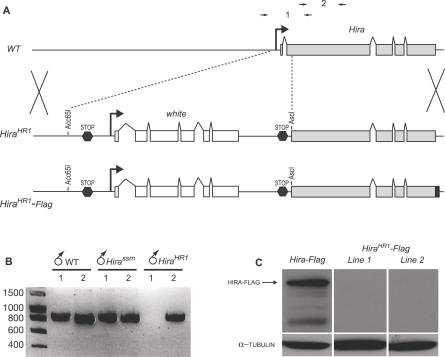
Targeting the *Hira* Gene by Homologous Recombination (A) Schematic representation of the wild-type (WT) *Hira* locus, the *Hira^HR1^* recombined allele, and the *pW25-Hira^HR1^-Flag* reporter transgene. The dotted lines indicate the region that is replaced by the *pW25* vector sequence in *Hira^HR1^*. The gray and white boxes indicate the *Hira* and *white* exons, respectively, and the black box is the *3X-Flag* tag at the 3' end of the *pW25-Hira^HR1^-Flag* transgene. The dark gray hexagons represent termination codons in the six reading frames. The positions of the primer pairs used in (B) are shown (arrows). (B) Example of a genomic PCR with the primer pairs shown in (A). Note that the primer pair #1 does not amplify the large *pW25* insertion in the *Hira^HR1^* allele. The tested male genotypes are indicated. (C) Anti-FLAG and anti-tubulin western blot analysis of embryo extracts from *Hira-Flag* and *Hira^HR1^-Flag* transgenic lines. The arrow indicates the HIRA-FLAG protein. Other smaller bands are interpreted as HIRA-FLAG degradation products.

The original *ssm^185b^* allele (referred to as *Hira^ssm^*) is a point mutation that replaces an evolutionary conserved arginine with a lysine (R225K) in the N-terminus region of HIRA [17]. This region is characterized in all HIRA proteins by the presence of a well-conserved domain containing seven WD-repeats. WD-repeats assemble into a structure called beta-propeller [21]. The *Hira^ssm^* mutation does not affect the normal recruitment of HIRA in the male nucleus at fertilization [17]. Nevertheless, it completely prevents the deposition of histones on paternal DNA [16,17], suggesting that the beta-propeller domain is important for the nucleosome assembly activity of HIRA. To gain insight into other possible functions of *Hira* not evident from the subtle *Hira^ssm^* mutation, we generated a new mutant allele using ends-out homologous recombination [22]. The targeting construct was designed to delete a 319 bp DNA fragment encompassing the complete predicted 5′ UTR, the first exon, the first intron, and the 5′ part of the second exon of *Hira*. In addition, the recombination arms used in this construct did not overlap any other predicted coding sequence, thus minimizing the risk of damaging adjacent genes. Finally, in the recombined allele, the 319 bp deletion was replaced with a 4778 bp sequence from the pW25 vector [23], containing the *white* marker gene flanked with stop codons in the six reading frames (Figure 1A). We recovered 59 independent recombination events on the X chromosome that did not complement the 100% female sterility associated with the *Hira^ssm^* mutation (Table 1). Surprisingly, all these lines produced viable and fertile mutant males. In all the lines that were further examined (*n* = 7), homozygous mutant females were also viable but produced embryos that never hatched (unpublished data). One line, named *Hira^HR1^* (homologous recombination 1), was arbitrarily chosen to conduct the rest of the analysis. The nature of the molecular lesion at the *Hira^HR1^* locus was verified by PCR analysis and sequencing of genomic DNA, and the expected recombination event was found, with no other detectable alteration (Figure 1B and unpublished data). We verified that the maternal effect phenotype associated with *Hira^HR1^* remained unchanged in hemizygous *Hira^HR1^/Df(1)ct4b1* females, *Df(1)ct4b1* being a large X chromosome deficiency that covers the *Hira* region [15]. In addition, the Hira^HR1^ phenotype was fully rescued by a single copy of a wild-type *Hira* transgene [17], demonstrating that no other important gene was affected by the *Hira^HR1^* recombination event (unpublished data).

**Table 1:**
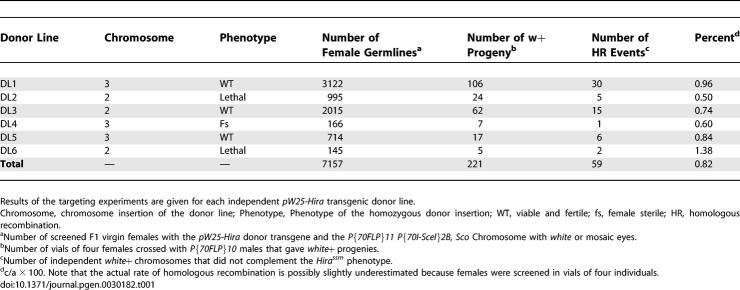
*Hira* Ends-Out Targeting

The *Hira^HR1^* mutation was expected to destroy the normal transcriptional regulation of *Hira*. However, transcriptional activity was detected by RT-PCR analysis at the junction between the pW25 vector and the beginning of the *Hira* sequence (unpublished data), suggesting that the *Hira^HR1^* allele could be transcribed from the hsp70 promoter associated with the *w^hs^* marker gene or from another promoter in or upstream from the pW25 vector. To check for the translation of any truncated HIRA protein from the *Hira^HR1^* allele, we first established transgenic lines containing a pW25-Hira^HR1^-Flag transgene (Figure 1A). This construct is identical to the donor transgene used for the homologous recombination with the exception of a 3X-Flag tag fused in frame to the C-terminus of HIRA. RT-PCR analysis of two independent pW25-Hira^HR1^-Flag lines confirmed that the *Hira* sequence in these transgenes is also transcribed (unpublished data). However, western-blot analysis of embryo extracts from both lines did not detect any HIRA-FLAG protein (Figure 1C).

**Figure 2:**
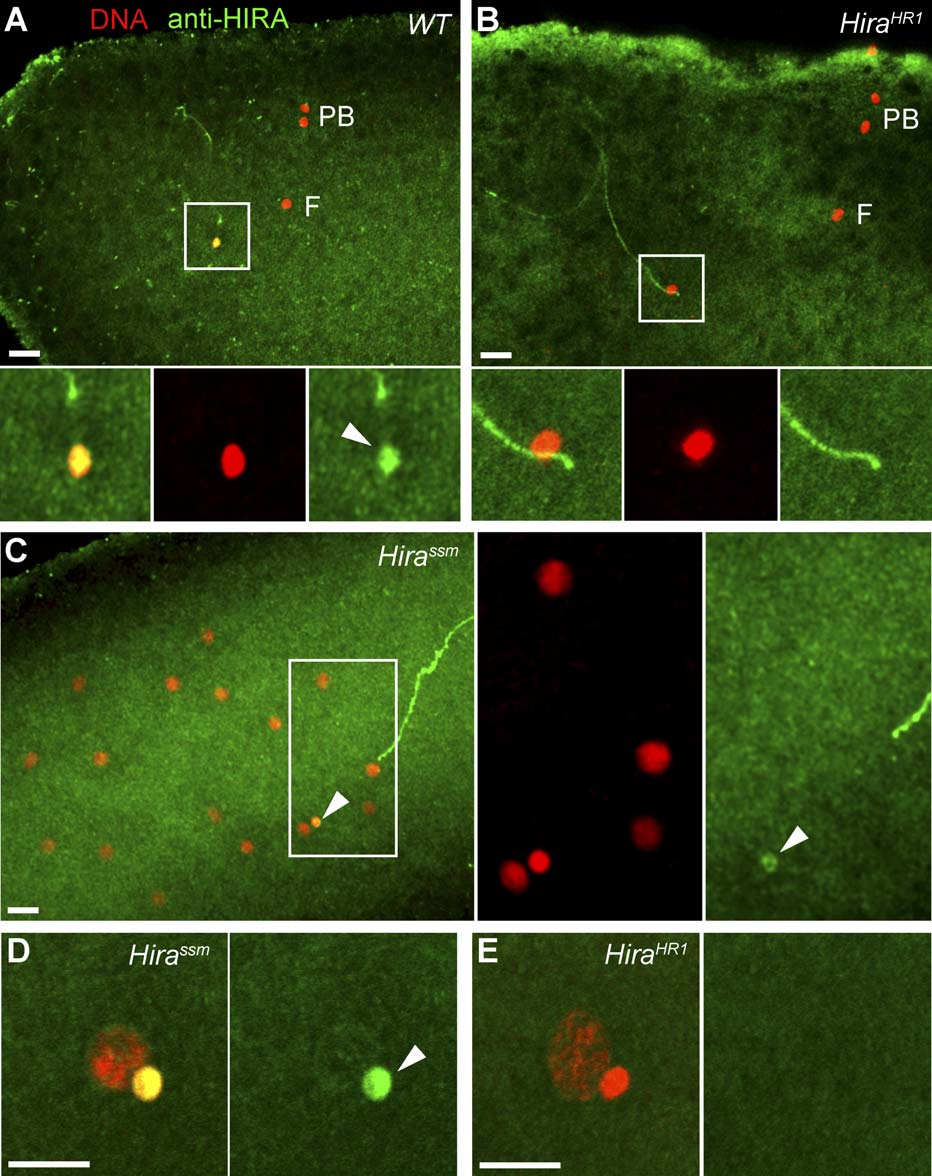
HIRA Is Not Detected in *Hira^HR1^* Eggs Confocal sections of eggs or embryos stained for DNA (red) and anti-HIRA antibodies (green). (A) In wild-type (WT) fertilized eggs, HIRA is specifically detected in the male nucleus (arrowhead in the inset). (B) In eggs from *Hira^HR1^* females, HIRA is not detected in the male nucleus (inset). Note that the HIRA antibody 830 non-specifically binds the sperm tail (elongated structure visible in the green channel) [17]. (C) A Cycle 5 haploid embryo from a *Hira^ssm^* female stained with antibody 830. The only stained nucleus is the condensed male nucleus (arrowheads). (D) Apposed pronuclei in a *Hira^ssm^* egg stained with HIRA antibody PG1 showing a strong signal in the male nucleus (arrowhead). (E) A *Hira^HR1^* egg at the same stage stained with the same antibody. F: Female pronucleus. PB: Polar Bodies. Bars: 10 μm.

We then directly tested the presence of HIRA in eggs from *Hira^HR1^* females using two independent HIRA polyclonal antibodies. The first antibody was raised against a mix of two synthetic HIRA oligopeptides [17] whose cognate DNA coding sequences are intact in the *Hira^HR1^* allele. The second antibody was raised against a recombinant protein containing residues 381–935 of HIRA (see Methods). Both sera readily detect maternal HIRA in wild-type and *Hira^ssm^* fixed eggs, as the protein specifically accumulates in the male pronucleus (Figure 2A, 2C, and 2D). As reported before [17], at the pronuclear apposition stage in *Hira^ssm^* eggs, the male pronucleus appeared much more condensed and smaller than the female pronucleus and brightly stained with anti-HIRA antibodies (Figure 2D). In *Hira^HR1^* eggs at the same stage, the male pronucleus looked identical to that in *Hira^ssm^* eggs, but did not contain any detectable HIRA protein (Figure 2B and 2E).

**Figure 3:**
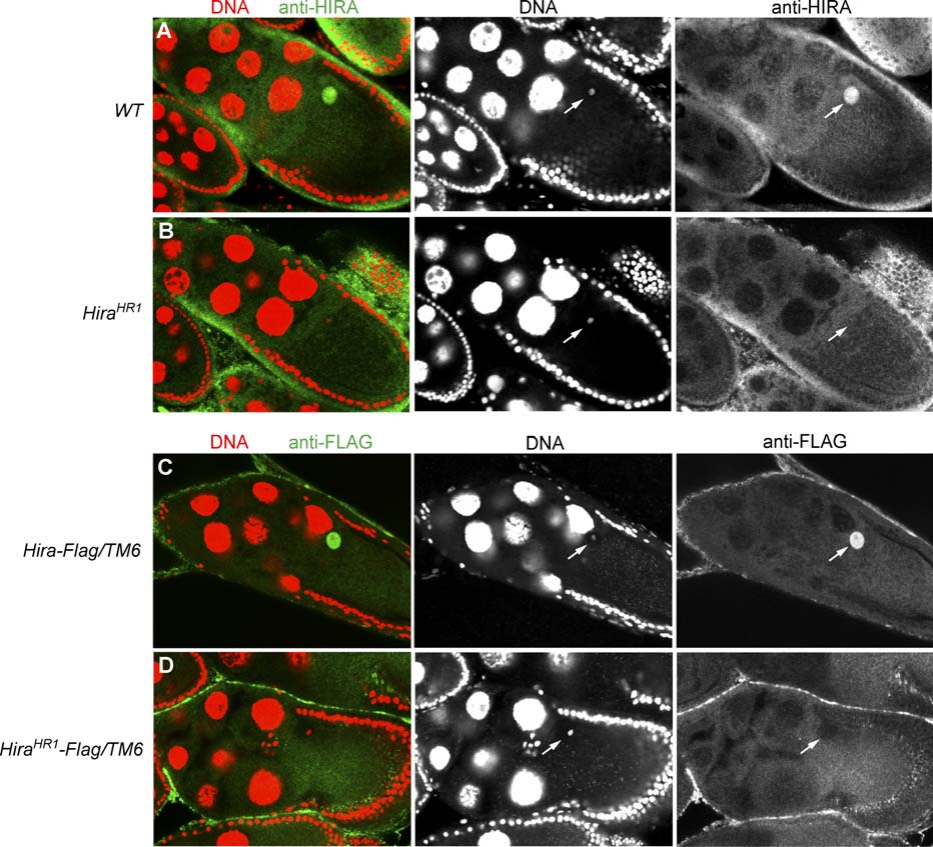
HIRA Accumulates in the Germinal Vesicle in Wild-Type but Not in *Hira^HR1^* Oocytes Stage 10 egg chambers stained for DNA (red) and anti-HIRA PG1 or anti-FLAG antibodies (green). (A) In wild-type egg chambers, HIRA is specifically detected in the germinal vesicle where it occupies the whole nuclear volume. The karyosome, the compact structure containing the maternal chromosomes, is visible in the DNA channel (arrow). (B) In *Hira^HR1^* egg chambers, the antibody does not detect HIRA in the germinal vesicle (arrow). (C) In transgenic *Hira-Flag* egg chambers, HIRA-FLAG protein is found in the germline vesicle (arrows) like the endogenous protein. (D) No HIRA-FLAG protein is detected in the oocyte nucleus in *Hira^HR1^-Flag* transgenic egg chambers.

Considering the fact that maternal HIRA protein is immediately available at fertilization to assemble paternal chromatin, we speculated that the protein must accumulate in growing oocytes during oogenesis. Indeed, wild-type ovaries stained with anti-HIRA antibodies revealed a specific signal in the oocyte nucleus (also called germinal vesicle) that was well visible from stage 10 of egg chamber formation (Figure 3A). The same staining of the oocyte nucleus was obtained with transgenic *Hira-Flag* ovaries stained with anti-FLAG antibodies (Figure 3C). Strikingly, the germinal vesicle staining was absent in *Hira^HR1^* ovaries and *Hira^HR1^-Flag* ovaries stained with anti-HIRA or anti-FLAG antibodies, respectively (Figure 3B and 3D). Altogether, these results strongly support the hypothesis that no HIRA protein is produced from the *Hira^HR1^* mutant allele.

### The Hira^HR1^ and Hira^ssm^ Phenotypes at Fertilization Are Indistinguishable

Previous studies of the *Hira^ssm^* allele had revealed that the male nucleus in mutant eggs was unable to undergo SCR [16]. Despite the fact that the mutant HIRA protein normally accumulates in the male nucleus in *Hira^ssm^* eggs ([17] and Figure 2D), it is unable to assemble chromatin. Consequently, the male nucleus does not achieve its decondensation and does not replicate its DNA.

**Figure 4:**
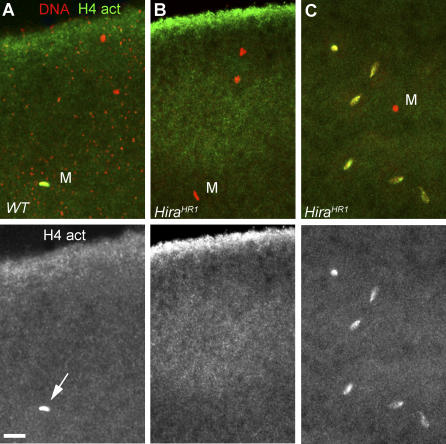
*Hira^HR1^* Eggs Are Unable to Assemble Paternal Chromatin at Fertilization Confocal sections of eggs and embryos stained for DNA (red) and anti-acetylated histone H4 antibody (green). (A) A wild-type egg in meiosis II with the elongated fertilizing male nucleus (M) that brightly stains for acetylated-H4 (arrow). (B) A *Hira^HR1^* egg at the same stage with no acetylated-H4 detected in the male nucleus. (C) A cycle 3 haploid embryo from a *Hira^HR1^* mother. The maternal nuclei, but not the male nucleus, stain for acetylated-H4. Bar: 10 μm.

At the cytological level, fertilized eggs from *Hira^HR1^* females appeared phenotypically identical to *Hira^ssm^* eggs. In all cases observed (*n* > 100), the male pronucleus remained abnormally small and condensed after pronuclear apposition (Figure 2E) and was unable to participate in the formation of the zygote (see Figure 4). As a consequence of this early defect, embryos from *Hira^HR1^* females were haploid, with only the maternal chromosome set.

To check for any RI nucleosome assembly in *Hira^HR1^* eggs, we used an anti-acetylated histone H4 antibody that brightly and specifically stains the decondensing male nucleus in wild-type eggs [17]. As expected, the massive RI nucleosome assembly that normally occurs during male pronucleus formation was not detected in *Hira^HR1^* eggs (Figure 4A and 4B). In contrast, RC deposition of acetylated H4 was normally detected in maternal nuclei (Figure 4C). Thus both *Hira^ssm^* and *Hira^HR1^* mutant alleles specifically prevent assembly of paternal chromatin and do not affect maternal nuclei.

### HIRA Is Not Involved in the Removal of Protamines from the Fertilizing Sperm Nucleus

**Figure 5:**
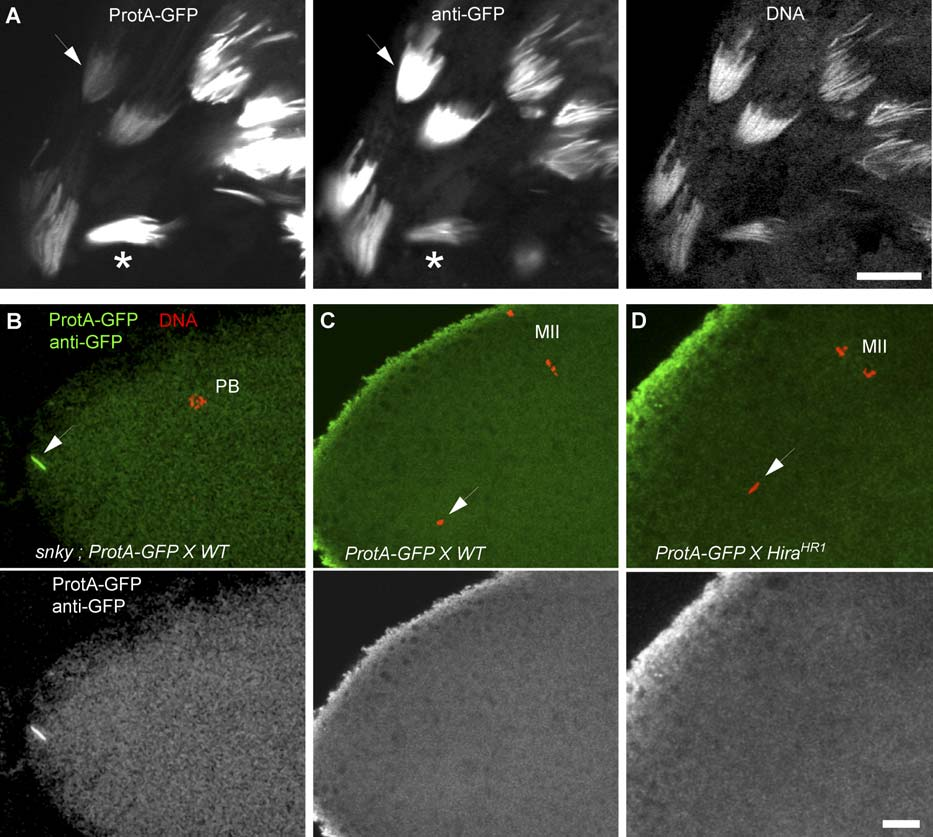
HIRA Is Not Required for Protamine Removal from the Decondensing Sperm Nucleus (A) Left panel: in a fixed *ProtA-GFP* transgenic testis, the GFP fluorescence is very strong in the most condensed spermatid nuclei (asterisk), whereas less condensed nuclei are much less bright (arrow). Middle panel: the same testis stained with an anti-GFP antibody considerably enhances the GFP detection in less condensed nuclei (arrow, compare with left panel), whereas highly condensed nuclei are comparatively less stained. Right panel: the same testis stained with the DNA dye TO-PRO3. (B) In wild-type (WT) eggs fertilized with sperm from *snky^1^ ; ProtA-GFP* males, the sperm nucleus is not activated (arrow), remains at the egg periphery, and its protamines are not removed. (C) In wild-type eggs fertilized with *ProtA-GFP* sperm and fixed before the end of meiosis II (MII), ProtA-GFP is never detected in the decondensing male nucleus (arrows). (D) The same result is obtained for *Hira^HR1^* eggs. Eggs in (B–D) were stained with an anti-GFP antibody revealed with a green secondary antibody to cumulate the GFP and secondary antibody respective fluorescence in the green channel of the confocal microscope. Identical results were obtained with ProtB-GFP transgenic males (unpublished data). PB: Polar Body. Bar: 10 μm.

In *Drosophila*, during spermiogenesis, post-meiotic spermatid nuclei progressively elongate and condense to eventually reach the typical needle-shape of mature sperm nuclei [24]. This complex process is also characterized by the replacement of histones with SNBPs, including two closely related protamines, ProtA and ProtB [13,14]. At fertilization, protamines are removed from the paternal chromatin, and nucleosomes are assembled in an RI process before the onset of the first zygotic S phase. The incapacity of the male nucleus to form in *Hira^ssm^* eggs led us to hypothesize that this phenotype could result from a defect in protamine removal [16]. Indeed, we would expect the persistence of protamines on paternal DNA to prevent nucleosome assembly and male nucleus decondensation. However, the presence of the HIRA protein in the male nucleus in *Hira^ssm^* eggs precluded drawing any conclusion about its role in protamine removal [13]. In contrast, the *Hira^HR1^* allele allowed us to address this point because in this case the protein is absent from the male nucleus. To document the dynamics of protamine removal at fertilization, we used transgenic males expressing ProtA-GFP or ProtB-GFP in their germ line [13]. These males are fertile and their testes contain groups of spermatid nuclei that achieve maximum fluorescence toward the end of the condensation process (Figure 5A, left panel). To verify that protamine-GFP can be detected in eggs, we crossed wild-type females with *ProtA-GFP* males homozygous for *sneaky* (*snky*), a paternal effect mutation that prevents sperm plasma membrane breakdown at fertilization and sperm activation [25]. We found that fertilizing sperm nuclei from *ProtA-GFP ; snky* males were brightly fluorescent in all cases observed (Figure 5B). We then looked at wild-type and *Hira^HR1^* eggs fertilized with *ProtA-GFP* or *ProtB-GFP* sperm. Even in the earliest eggs we observed, we never detected any trace of Prot-GFP in the decondensing male nucleus (Figure 5C and 5D). We thus concluded that the removal of protamines from the fertilizing sperm nucleus is a fugacious, HIRA-independent process that must occur immediately after sperm plasma membrane breakdown and before the onset of the second meiotic division.

### RI Paternal Chromatin Assembly Does Not Depend on Egg Activation

**Figure 6:**
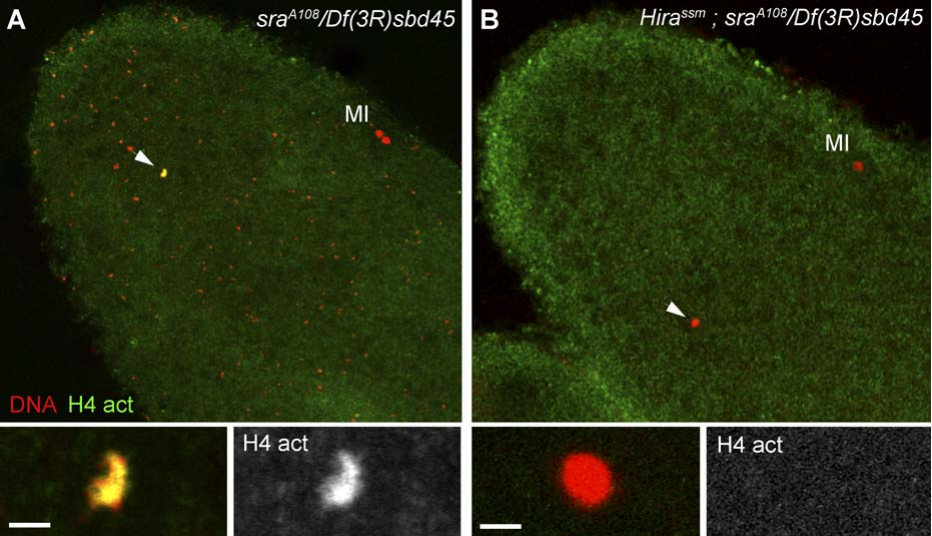
The Male Nucleus Does Not Recondense in *Hira ; sra* Double Mutant Eggs (A) In *sra^A108^/Df(3R)sbd45* mutant eggs, the female meiosis arrests in anaphase of the first meiotic division (MI). The male nucleus (arrowhead and bottom panels) appears condensed but irregular in shape and stains with anti-acetylated histone H4 antibodies (bottom right panel). Note that the DNA positive dots that are visible in this egg are *Wolbachia* bacteria that naturally infect the stock. (B) In *Hira^ssm^ ; sra^A108^/Df(3R)sbd45* Double Mutant Eggs, the Male Nucleus Is Round and Does Not Stain with Anti-Histone Antibodies. Bars: 2 μm.

In *Drosophila*, mature oocytes are arrested in metaphase of the first meiotic division until egg ovulation and activation. In contrast to many animals, egg activation in flies is not dependent on fertilization. Instead, eggs are reactivated during ovulation and immediately resume meiosis [26]. *Drosophila* females with a mutated *sarah* (*sra*) gene lay eggs that are defective in several aspects of egg activation, including a meiotic block in anaphase of the first division [27]. Interestingly, these authors observed that the male pronucleus in fertilized *sra* eggs remained abnormally condensed and did not replicate its DNA. This aspect of the sra phenotype presents striking similarities with the Hira mutant phenotype, raising the possibility that HIRA activity could depend on egg activation. In their paper, Horner et al. observed that the male nucleus and maternal chromosomes stained, although rather diffusely, with an anti-histone H1 antibody. They concluded that paternal chromatin remodelling was not impaired in *sra* eggs. However, it has been previously reported that early *Drosophila* embryos lack histone H1 [28], opening the possibility that anti-H1 antibodies could cross-react with a non-H1 epitope. To directly analyse paternal chromatin assembly in *sra* eggs, we used anti-acetylated-H4 antibodies. In all cases, the condensed male nucleus, but not the maternal chromosomes, brightly stained with the anti-acetylated-H4 antibody, confirming that paternal chromatin assembly is not dependent on egg activation (Figure 6A). In addition, we verified that ProtA-GFP was not detected from the male nucleus in *sra* eggs fertilized with *ProtA-GFP* males (unpublished data).

In *sra* eggs blocked in anaphase of the first meiotic division, the male nucleus frequently presented a rather irregular shape (Figure 6A) and an apparent level of DNA condensation that was comparable with the highly condensed maternal chromosomes blocked in anaphase I of the first meiotic division. Hence, the high level of cyclin B in *sra* eggs that causes the meiotic block [27] could also affect the male nucleus and force it to recondense its unreplicated chromatin. In comparison to *sra*, the male nucleus in *Hira^ssm^* mutant eggs is a uniformly round nucleus that systematically adopts its definitive shape by the end of female meiosis II [17]. To see if the *Hira^ssm^* male nucleus could recondense in *sra* eggs, we constructed double mutant *Hira^ssm^*/*Hira^ssm^ ; sra^A108^/Df(3R)sbd45* females. In fertilized eggs from these double mutant females, we observed that the male nucleus did not stain with anti-acetylated-H4 antibodies and looked identical in shape and size to the male nucleus in *Hira^ssm^* eggs (Figure 6B). Thus, in the absence of an assembled chromatin, the male nucleus is unable to recondense in response to the meiotic block of *sra* eggs.

### The ASF1 Histone Chaperone Is Not Involved in the RI Assembly of Paternal Chromatin

**Figure 7:**
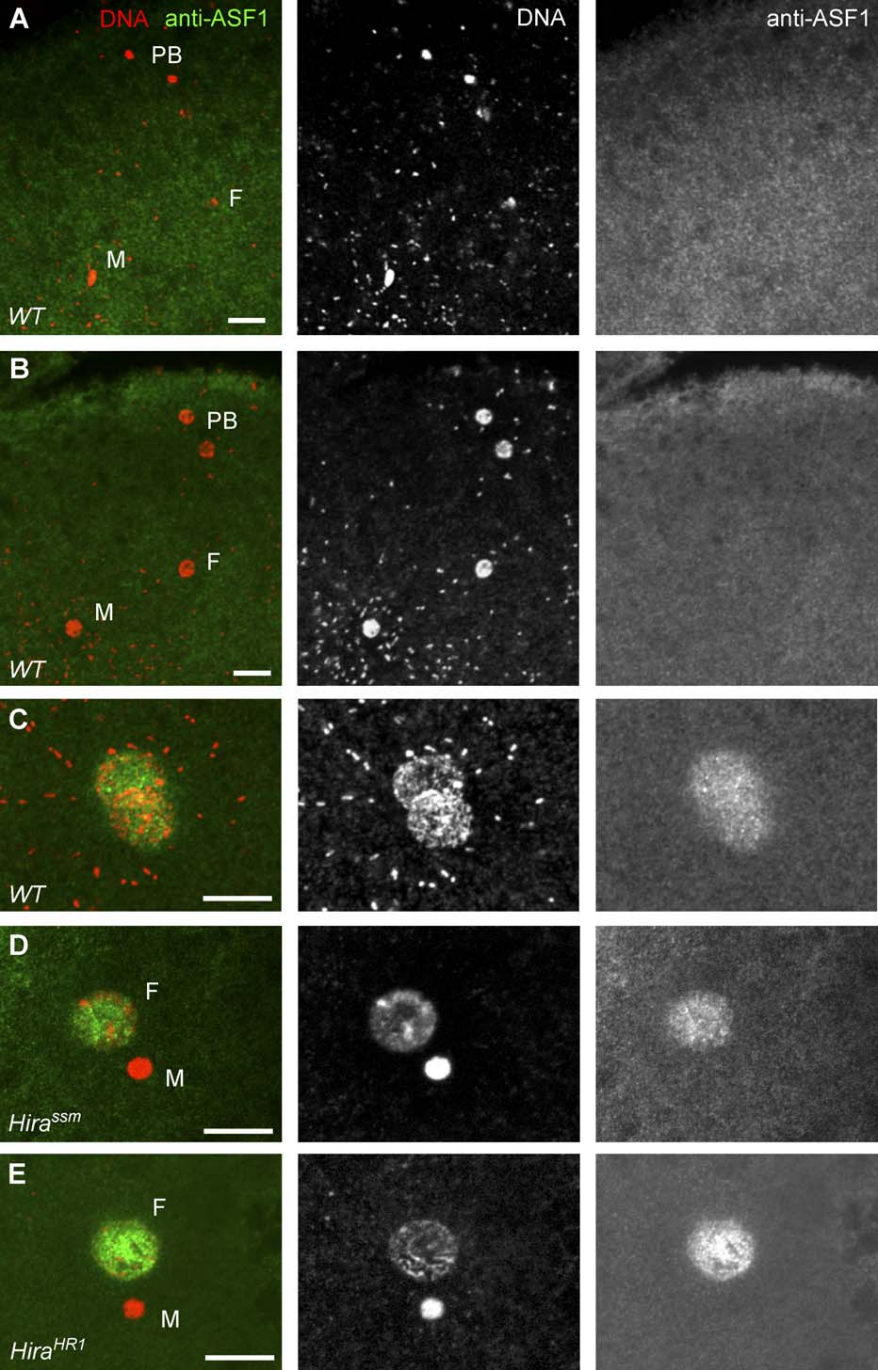
ASF1 Is Not Directly Involved in the RI Paternal Chromatin Assembly Confocal sections of eggs stained for DNA (red) and anti-ASF1 antibody (green). (A) In wild-type fertilized eggs, ASF1 is not detected in the male nucleus or in maternal nuclei during the decondensation phase. (B) ASF1 is not detected in the male nucleus during pronuclear migration. (C) ASF1 stains both pronuclei in a wild-type egg during the first S phase. (D) ASF1 is not detected in the male nucleus in *Hira^ssm^* eggs. (D) The same result was obtained with the *Hira^HR1^* allele. F: Female pronucleus, M: Male pronucleus, PB: Polar Bodies. Bar: 10 μm.

SCR provides a unique opportunity to study de novo nucleosome assembly in vivo at the scale of a whole nucleus and in the absence of DNA synthesis or transcription. A striking feature of this process is the very specific use of the H3.3 histone variant to assemble paternal nucleosomes, despite the presence of large quantities of canonical H3 stored in the egg cytoplasm. ASF1 is a conserved histone chaperone involved in the assembly of chromatin during DNA replication (reviewed in [29]). Recent studies have shown that ASF1 specifically interacts with H3-H4 dimers [30,31] and with HIR proteins [32,33], and could play a key role in presenting dimers containing specific H3 variants to their corresponding chaperones, such as H3 to CAF-1 and H3.3 to HIRA [29,31,33]. Accordingly, ASF1 proteins are found in both H3.1 and H3.3 complexes in human cells [19]. To investigate this possibility in our model, we stained fertilized eggs with an antibody against the unique *Drosophila* ASF1 protein [34]. We observed that ASF1 was systematically detected in replicating nuclei, including the pronuclei (Figure 7C). However, ASF1 was not found on the decondensing male nucleus in wild-type eggs or in the male nucleus in *Hira* mutant eggs (Figure 7A, 7B, 7D, and 7E). Thus, ASF1 does not directly cooperate with HIRA during the RI assembly of paternal chromatin. This is consistent with a recent report showing that ASF1 is dispensable for direct de novo histone deposition in Xenopus egg extracts [35]. So far, HIRA is the only H3-H4 chaperone involved in SCR in vivo.

### H3.3 Deposition Is Not Globally Affected in *Hira^HR1^* Mutant Embryos and Adults

**Figure 8:**
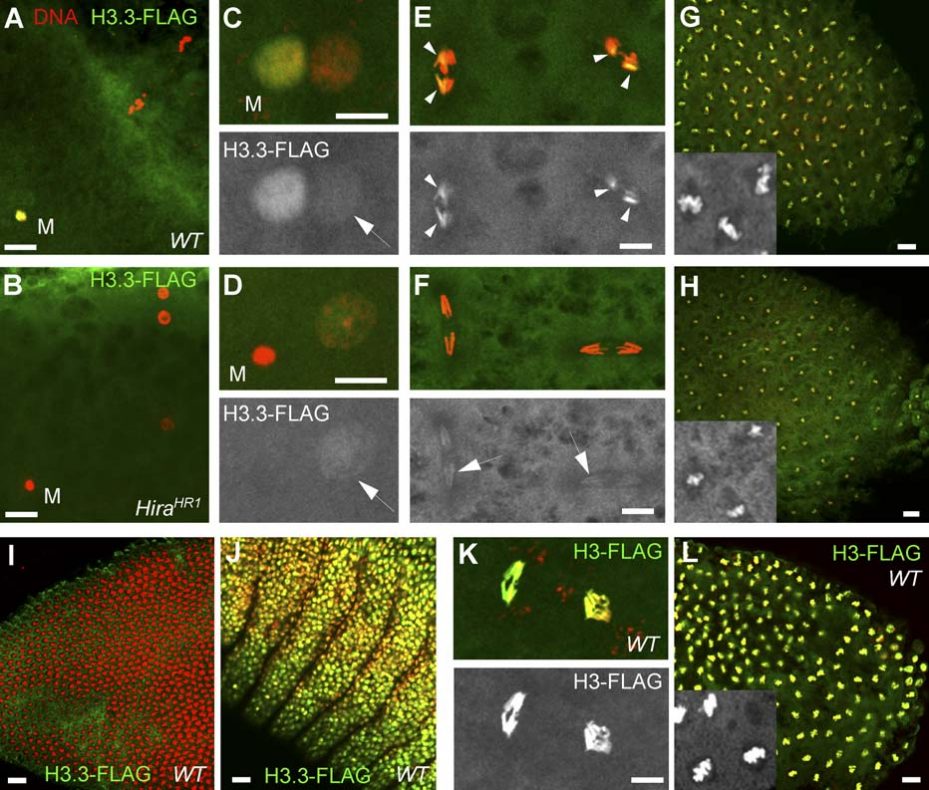
Dynamics of H3.3 Deposition in Wild-Type and *Hira^HR1^* Early Embryos Confocal sections of eggs/embryos stained with propidium iodide (red) and anti-FLAG antibody (green). (A) In wild-type (WT) eggs, RI deposition of maternal H3.3-FLAG is observed in the decondensing male nucleus (M) before the first zygotic S phase. (B) H3.3-FLAG is not detected in the male nucleus in *Hira^HR1^* eggs. (C) At pronuclear apposition, during the first S phase, limited RC deposition of H3.3-FLAG is detected in the female pronucleus (arrow) in WT eggs. (D). The same, faint H3.3-FLAG staining of the female pronucleus is observed in *Hira^HR1^* eggs (arrow)*.* (E) A WT embryo in anaphase of the third nuclear division. At this early stage, the stronger H3.3-FLAG staining of the paternally derived chromosomes (arrowheads) is still detectable (note that paternal and maternal chromosomes tend to remain separated during the early syncytial mitoses). (F) A *Hira^HR1^* haploid embryo in its fourth mitosis showing a weak H3.3-FLAG staining on maternally derived chromosomes (arrows). (G) A wild-type, diploid blastoderm embryo in metaphase showing a strong H3.3-FLAG chromosomal staining on all nuclei. (H) H3.3-FLAG is also detected on the chromosomes of *Hira^HR1^* haploid blastoderm embryos. (I) Embryos from wild-type mothers crossed with *H3.3-Flag/CyO* males showing no detection of zygotic H3.3-FLAG at this stage. (J) Zygotic H3.3-FLAG appears in the chromatin of gastrula embryos. (K) A wild-type, cycle 3 embryo in anaphase showing a strong H3-FLAG staining on all chromosomes. (L) A blastoderm embryo with a strong maternal H3-FLAG staining. Gray panels or insets show the H3.3-FLAG or H3-FLAG staining for a representative group of nuclei. Bar: 10 μm.

The analysis of the *Hira^HR1^* allele confirmed the essential role of maternal HIRA for the RI chromatin assembly in the male pronucleus. In *Drosophila*, early development is under maternal control and zygotic transcription essentially begins at the blastoderm stage [26]. In embryos, HIRA antibodies did not produce any detectable staining, suggesting that the protein, if it plays any role, does not accumulate at high levels in embryo nuclei like in the male pronucleus (unpublished data). Haploid embryos laid by *Hira^HR1^* females (named *Hira^HR1^* embryos for simplicity) arrest their development just before hatching. We used this situation to study H3.3 deposition in wild-type and *Hira^HR1^* early embryos. We used a previously described transgenic line expressing H3.3-FLAG under the regulatory sequences of the *Drosophila His3.3A* gene [17]. Maternally expressed H3.3-FLAG was then revealed using anti-FLAG antibodies. Zygotically expressed H3.3-FLAG becomes detectable in chromatin only at the gastrula stage (Figure 8I and 8J) and was thus not detected in our experiments on early embryos. As reported before [17], in wild-type eggs, H3.3-FLAG is first detected in the decondensing male nucleus shortly after fertilization (Figure 8A). As expected, the male nucleus does not contain any H3.3-FLAG in *Hira^HR1^* eggs, confirming the absence of chromatin assembly in the male nucleus (Figure 8B). At the pronuclear apposition stage in wild-type eggs, after the first round of DNA replication, H3.3-FLAG is still abundant in the male nucleus, but a faint staining is also visible in the female pronucleus (Figure 8C) and polar bodies (unpublished data). Interestingly, this H3.3-FLAG staining in the female pronucleus is also detected in *Hira^HR1^* eggs at the same stage (Figure 8D). H3.3 can be deposited on DNA through a transcription-coupled (TC) assembly mechanism, suggesting that the passage of the RNA polymerase complex displaces nucleosomes and creates a need for RI assembly [36]. In the absence of transcription in early *Drosophila* embryos, the observed H3.3-FLAG must occur through a transcription-independent process, presumably during DNA replication. In wild-type embryos, we observed that the initial enrichment of H3.3-FLAG on paternal chromosomes was still detectable during the first 3 or 4 nuclear cycles (Figure 8E). In *Hira^HR1^* early embryos, only a faint H3.3-FLAG staining was detected on the sole maternally derived set of chromosomes (Figure 8F). The paternal H3.3 mark in wild-type embryos was no longer detectable in later embryos (unpublished data) suggesting a rapid dilution by the massive RC deposition of H3 that occurs at each S phase. To verify this point, we used a transgenic line that expresses H3-FLAG with the regulatory sequences of *His3.3A* [17]. Both H3-Flag and H3.3-Flag transgenes produce equivalent levels of tagged histones in embryos [17] and allow a direct comparison of their respective deposition during early development. During the earliest mitoses, the H3-FLAG staining on chromosomes was much stronger than the H3.3-FLAG staining (Figure 8K, compare with Figure 8E), confirming that H3 is much more efficiently incorporated in chromatin than H3.3 at this stage. The difference between H3.3-FLAG and H3-FLAG chromosome staining was also visible in blastoderm embryos (Figure 8G and 8L). At the blastoderm stage, H3.3-FLAG clearly marked the chromatin of all nuclei in both WT and *Hira^HR1^* (Figure 8G and 8H). In conclusion, although H3 is preferentially deposited during the early nuclear cycles, our results demonstrate that H3.3 is also deposited at this stage, through a HIRA-independent assembly pathway. Further work will be required to determine whether this HIRA-independent H3.3 deposition occurs during or independently of DNA replication.

**Figure 9:**
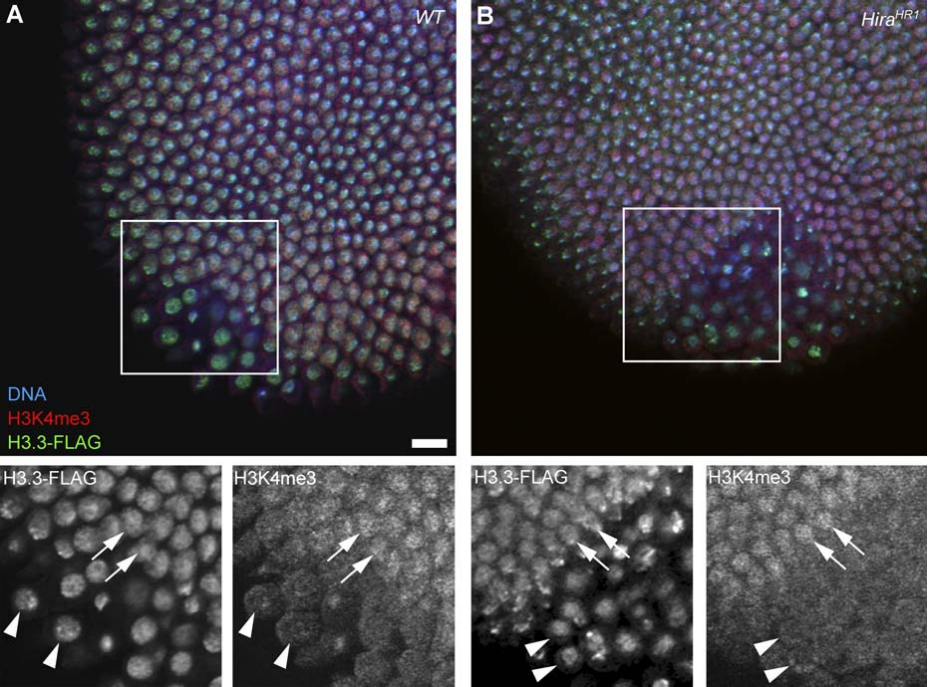
H3.3-FLAG Is Deposited in the Germ Line Chromatin in Blastoderm Embryos Confocal sections of blastoderm embryos stained for DNA (blue), H3.3-FLAG (green), and H3K4me3 (red). (A) H3.3-FLAG (left inset) is deposited at equivalent levels in somatic (arrows) and germ line (arrowheads) nuclei in wild-type embryos. H3K4me3 is enriched in somatic nuclei (right inset). (B) An identical situation is observed in *Hira^HR1^* embryos. Bar: 10 μm.

The migration of nuclei at the embryo periphery correlates with the onset of zygotic transcription, with the notable exception of germ line pole cells that are kept silent until stage 9/10 of embryo development [37]. Interestingly, we observed that H3.3-FLAG is deposited at equivalent levels in somatic and in pole cell nuclei in both wild-type and *Hira^HR1^* embryos (Figure 9). Thus, TC assembly does not seem to contribute substantially to the observed level of H3.3-FLAG in chromatin at this stage. The activation of the zygotic genome in blastoderm embryos correlates well with the apparition of histone post-translational modifications associated with transcriptionally active chromatin, such as the methylation of histone H3 at lysine 4 [38]. Figure 9 shows that this active mark is normally detected in *Hira^HR1^* embryos, suggesting that HIRA is not required for the remodelling of chromatin associated with the onset of zygotic transcription. Accordingly, *Hira^HR1^* embryos develop without obvious problems until late embryogenesis and eventually arrest development with a phenotype typical of haploid embryos produced by other mutants ([39,40] and unpublished data).

**Figure 10:**
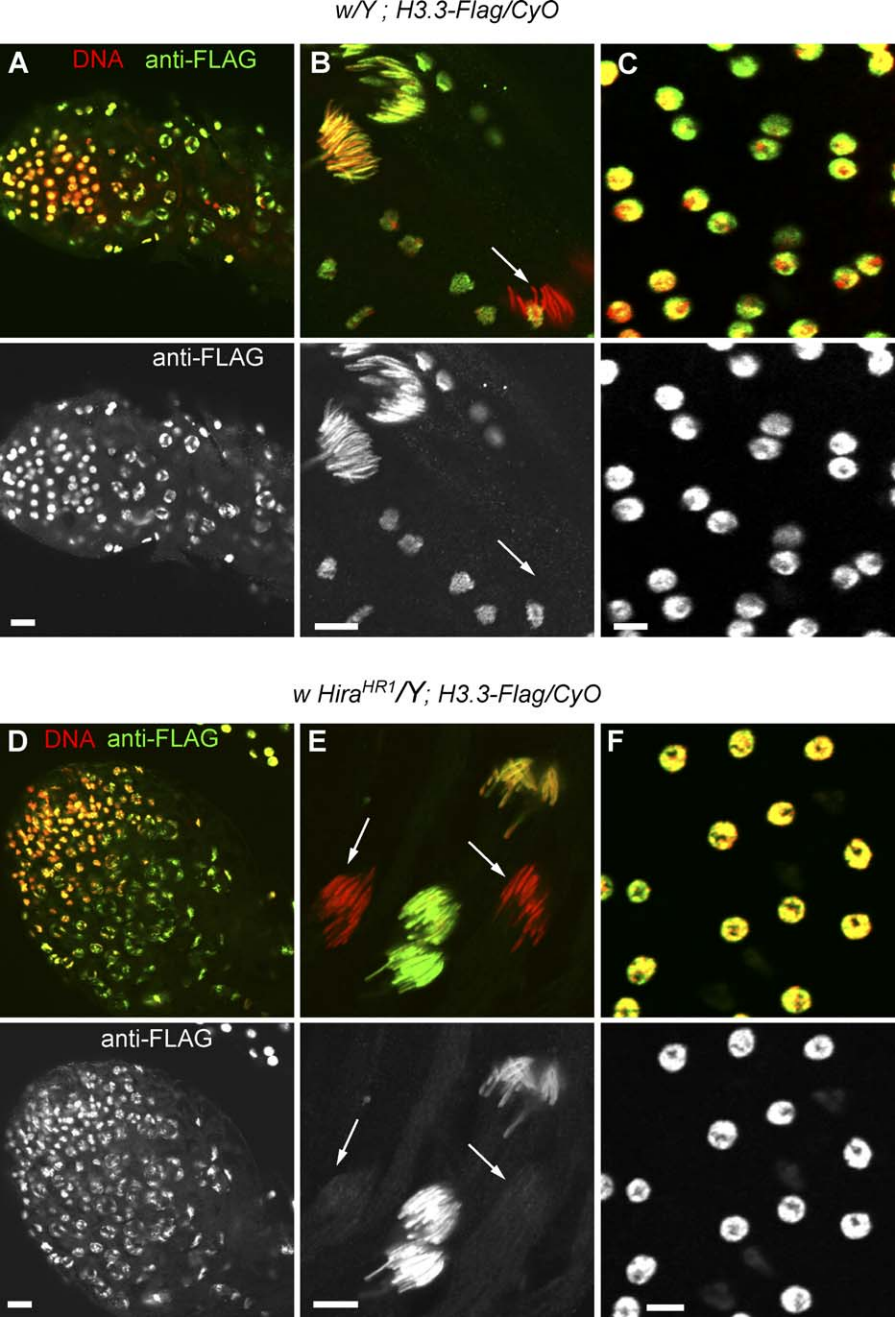
*Hira^HR1^* Does Not Affect the Distribution of H3.3-FLAG in Adult Testis Testis and accessory glands from *H3.3-Flag/CyO* transgenic adult males with a wild-type (A–C) or *Hira^HR1^* (D–F) X chromosome, stained with anti-FLAG antibody and propidium iodide. (A) Apical tip of a wild-type testis. (B) A group of elongating spermatids in a wild-type testis showing a bright H3.3-FLAG nuclear staining that disappears in late condensing spermatid nuclei (arrow). (C) Nuclei from a wild-type accessory gland. (D) Apical tip of a *Hira^HR1^* testis. (E) Spermatid nuclei in a *Hira^HR1^* testis. H3.3-FLAG is not detected in late spermatid nuclei (arrows). (F) Nuclei from a *Hira^HR1^* accessory gland. Bars: 10 μm.

That *Hira^HR1^* flies are viable offered us the possibility to evaluate the impact of the mutation on H3.3-FLAG distribution in adult tissues. We chose to focus on the testis, an organ where H3.3 distribution had been characterized already [41]. In wild-type transgenic adult testis, we observed a strong nuclear staining of H3.3-FLAG in all somatic and germline nuclei with the exception of late spermatid and sperm nuclei, similar to previous reports [41]. In *Hira^HR1^* testis we found no detectable alteration of the distribution of H3.3-FLAG in both somatic and germ line nuclei (Figure 10). We then looked at other adult tissues including ovaries, malpighian tubules, and gut; again, we found no difference between control and mutant (unpublished data). We conclude that, with the sole exception of the male pronucleus, HIRA does not seem to play any crucial role for the assembly of H3.3 nucleosomes during *Drosophila* development.

## Discussion

### HIRA and SCR

The analysis of maternal effect mutations in the *Drosophila Hira* gene has revealed that SCR at fertilization involves at least two functionally distinct steps. The first step is a HIRA-independent process that allows the rapid removal of protamines from the activated sperm nucleus. The second step is the RI nucleosome assembly on paternal DNA and requires maternal HIRA. That the male pronucleus seems to be the only nucleus where H3.3 deposition is critically dependent on HIRA (see below) indicates a peculiar case of RI assembly. This could reflect specific features of the sperm nucleus itself or constraints inherent to the tightly time-controlled, whole paternal genome assembly at fertilization. At least we know that this specific requirement of HIRA for SCR is not directly linked to the removal of protamines.

Our finding that SNBP removal activity is functionally uncoupled to nucleosome assembly in *Drosophila* does not apply to all known cases of SCR in animals. In fact, in the classical example of SCR in Xenopus laevis, it was demonstrated through in vitro experiments that a unique histone chaperone, nucleoplasmin, was necessary and sufficient to perform both SNBP removal and histone deposition [42,43]. Nucleoplasmin is a small, acidic protein that is highly abundant in amphibian oocytes and forms pentameric complexes that associate with core histones [2,44,45]. It is important to consider, however, that the protein composition of *Xenopus* sperm chromatin is rather peculiar since it essentially retains H3-H4 tetramers on paternal DNA, whereas H2A and H2B are replaced with protamine-like proteins named SPs [43,46]. In vitro, nucleoplasmin allows the replacement of SPs with H2A and H2B and reconstitute nucleosomes [43,44]. There is apparently no need for a H3-H4 assembly factor such as HIRA for *Xenopus* SCR. A nucleoplasmin-like protein exists in *Drosophila*, but studies of its ability to decondense demembranated *Xenopus* sperm nuclei in vitro have led to contradictory results [11,12]. The actual function of *Drosophila* nucleoplasmin remains to be determined. In addition, other *Drosophila* embryonic nuclear factors are known to decondense *Xenopus* sperm in vitro, such as DF31 [10] and NAP-1 [11], but their protamine removal activity has not been confirmed in vivo. In mouse, as in *Drosophila*, sperm chromatin is essentially packaged with protamines [47]. Interestingly, the knock-out of *NPM2*, the mouse ortholog of *Xenopus* nucleoplasmin, does not affect SCR [48]. In contrast, HIRA is very likely involved in the assembly of paternal chromatin in the mouse zygote. Indeed, in this species, HIRA is detected in the decondensing male nucleus [49] and H3.3 is specifically deposited on paternal DNA in an RI manner [49,50]. We thus expect HIRA to be generally involved in the assembly of paternal chromatin in animal species in which histones H3 and H4 are totally or partially replaced with SNBPs in the mature sperm. As an H3.3-H4 deposition factor, HIRA itself is not expected to mediate the deposition of H2A-H2B required for the completion of nucleosome assembly on paternal DNA. It will be interesting to identify this H2A-H2B chaperone and see if it is dedicated to RI assembly or involved in both RI and RC assembly pathways.

In *Hira* mutant eggs, the male nucleus is a small, round nucleus that appears homogeneously condensed when stained with a DNA dye. How the paternal DNA is organised in this nucleus is not known. That it is surrounded by a de novo assembled nuclear lamina [16] probably participates in the maintenance of its round shape. Also, it is established that the four centromeric regions are the only regions that are organized with histones, most likely because centromeric chromatin is not replaced with protamines in the sperm nucleus [16]. In this paper, we have demonstrated that the male nucleus in *Hira* mutant eggs is also devoid of protamines, strongly suggesting that most paternal DNA is free of chromosomal proteins. A similar situation was reported in decondensation assays using sperm from Bufo japonicus, a toad species whose sperm chromatin only contains protamines [51]. In the presence of nucleoplasmin, protamines are efficiently removed but nucleosomes are not assembled. Consequently, B. japonicus sperm nuclei decondensed with egg extracts containing the protamine removal activity possess neither protamines nor core histones, and are very fragile [51]. Similarly, in *Hira* mutant eggs, the removal of protamines from the male nucleus permits its partial decondensation as the sperm nuclear volume increases when the nucleus loses its specific needle shape and becomes round. However, in the absence of a nucleosomal organisation, the male nucleus cannot achieve its decondensation and does not replicate its DNA. This unique, inert state of the male nucleus in *Hira* mutant eggs is also well illustrated by its incapacity to recondense in blocked *sra* mutant eggs.

### The Function of HIRA during *Drosophila* Development

A surprising aspect of this study is the viability of *Hira^HR1^* homozygous flies. This was unexpected, because in mouse the *Hira* knock-out is embryonically lethal [52]. From a genetic point of view, both *Hira^ssm^* and *Hira^HR1^* alleles behave as null alleles with respect to the *Df(1)ct4b1* deficiency. In addition, several lines of evidence indicate that no HIRA protein is translated in *Hira^HR1^* flies, including the absence of detection of HIRA in the germinal vesicle and the male pronucleus, and the absence of HIRA-FLAG protein expressed from the *pW25-Hira^HR1^-Flag* reporter transgene. In the alternative possibility that some truncated HIRA protein would be translated from this allele and escaped our detection, the first possible translation initiation codon downstream from the deleted region in *Hira^HR1^* is at position 61, after the second WD repeat. Such a truncated HIRA would thus be expected to have, at best, a destabilized beta-propeller domain, which represents the most evolutionarily conserved part of HIRA proteins [53,54]. The fact that both *Hira^ssm^* and *Hira^HR1^* alleles display identical mutant phenotypes also highlights the very important role of the arginine 225 mutated in *Hira^ssm^*, and by extension, the important role of the beta-propeller domain for the assembly of paternal chromatin. A recent study implicated *Drosophila* HIRA and the GAGA factor–FACT complex in a histone replacement mechanism that prevents the spreading of heterochromatin into a *white* reporter transgene inserted near centromeric heterochromatin [20]. Nakayama et al. observed that silencing of this variegating transgene was enhanced in *Hira^ssm^* males, and concluded that the mutation affected H3.3 replacement at a site near the *white* gene. Their work suggests that *Drosophila* HIRA could indeed function in RI assembly in other situations and is consistent with the fact that *Hira* is expressed throughout development, in addition to its strong maternal expression [17,53,54]. Nevertheless, the fact that *Hira^HR1^* mutant adults are viable indicates that this function is dispensable.

### H3.3 Deposition without HIRA

Another important aspect of this study lies in the fact that the *Hira^HR1^* mutation does not have detectable effect on the deposition of H3.3-FLAG in embryos or adult cells. First, it clearly establishes that H3.3 nucleosomes can be efficiently assembled in the absence of functional HIRA in vivo. So far HIRA is the only chaperone known to deposit the H3.3 variant. This study demonstrates the existence of at least one alternative assembly pathway for H3.3 nucleosomes, although the nature of the histone chaperone(s) involved is unknown. A simple hypothesis is the deposition of H3.3 by the CAF-1 complex. In fact, we have shown that in early embryos, the bulk of H3.3 is deposited independently of transcription, presumably at each S phase of the early nuclear cycles. Indeed, these cycles consist on a very rapid succession of S and M phases and lack gap phases [26]. The S phase deposition of H3.3 is consistent with a previous report showing that overexpressed H3.3-GFP was deposited during DNA replication in *Drosophila* Kc cells [55]. In human cells, only the small subunit of CAF-1 was found in the H3.3 complex, whereas all three subunits of the complex were copurified with the replicative histone H3.1 [19]. In early cycles, H3 is preferentially deposited compared with H3.3. However, a peculiarity of *Drosophila* embryos is the storage of large maternal pools of both H3 and H3.3, a situation that could favour a competition of these histones for their interaction with CAF-1. In contrast, in differentiated cells, the massive expression of S phase histones at the onset of DNA replication could strongly reduce the use of H3.3-H4 dimers by the CAF-1 complex. The early *Drosophila* embryo should represent a good model to address this point.

A study of *Hira* −/− mouse ES showed that these cells undergo early differentiation, suggesting that core histone deposition during this process could use HIRA-independent pathways [56]. Although it is well established that H3.3 deposition correlates with active chromatin in many instances, there is yet no link between HIRA and transcription in higher eukaryotes [57]. In budding yeast, nucleosome reassembly at the PHO5 promoter absolutely requires the histone H3-H4 chaperone Spt6 [58], whereas Hir1 is not absolutely required [59,60]. In *Drosophila*, Spt6 is clearly involved in transcription elongation [61,62] and thus represents an interesting candidate for TC deposition of H3.3 [57]. The biochemical analysis of H3.3 complex in *Hira^HR1^* mutant could help identify alternative H3.3 chaperone(s).

Our results support the hypothesis that multiple and possibly redundant pathways are involved in the assembly of H3.3 nucleosomes in multicellular organisms. Besides, it is now established that H3.3 nucleosomes can be assembled independently of RC and TC assembly pathways. For example, nucleosome replacement mechanisms at cis-regulatory elements implicating the deposition of H3.3 have been recently reported in *Drosophila* [20,63]. The ability of cells to assemble chromatin independently of DNA replication is apparently common to all eukaryotes. In fact, some organisms such as yeasts have only one type of histone H3, which is related to H3.3 and is deposited throughout the cell cycle [64]. The coexistence of RC and RI histone H3s in most other eukaryotes indicates that these distinct modes of chromatin assembly fulfil important complementary functions. Interestingly and surprisingly, the deletion of all RI H3 histone genes in the protist Tetrahymena thermophila does not compromise survival and, in particular, does not affect nucleosome density at highly transcribed regions [65]. However, RI H3 genes in T. thermophila appear to be critical for the production of viable sexual progeny and for the function of germline micronuclei [65], suggesting that sexual reproduction and/or developmental processes could have played an important role in the evolution of the RI mode of nucleosome assembly. RI H3.3 replacement at fertilization is apparently a conserved mechanism in nematodes, insects, vertebrates, and plants [17,49,50,66,67]. That the paternal chromatin assembly is the only essential function of *Drosophila* HIRA suggests that this factor acquired new important roles during the evolution of vertebrates. So far, in mammals, the implication of the HIRA/H3.3 complex has been shown or at least suspected in various remodelling processes, including heterochromatin repair [68], mammalian meiotic sex chromosome inactivation [69], fertilization [49,50], and possibly, formation of senescence-associated heterochromatin foci [70] and histone exchange during spermiogenesis [71]. More functional studies should reveal if all these processes strictly rely on HIRA, in the context of the developing organism.

Note: After the preparation of our manuscript, a paper by A. Konev et al. [72] was published that reported the implication of the motor protein CHD1 in the deposition of histone H3.3 in *Drosophila*. This finding supports our own conclusions about the existence of Hira-independent H3.3 deposition pathways.

## Materials and Methods

### Flies.

The *w^1118^ ssm^185b^*/FM7c stock was described before [15]. The ProtamineA/B-GFP stocks [13] are a gift from S. Jayaramaiah Raja and R. Renkawitz-Pohl. The *sra^A108^* allele [27] is a gift from V. Horner and M. Wolfner. Df(3R)sbd45 is a deficiency that covers the *sra* locus. The H3.3-Flag, H3-Flag, and *Hira*-Flag stocks have been described before [17]. The *y w^67c^* and *w^1118^* stocks were used as wild-type controls. All the other stocks or chromosomes used in this paper were obtained from the Bloomington *Drosophila* stock center.

### *Hira* targeting by homologous recombination.

The *Hira* gene was targeted by ends-out homologous recombination as described in [22,23]. Two DNA fragments from the *Hira* locus were PCR-amplified from the cosmid genomic DNA clone 107B5 (European *Drosophila* Genome Project) using the following primers: 5′-ATGAAATGAGTGCCAGCAGC-3′ and 5′-GGTACCTATCGGTAACGATGCCCATC-3′ for the *Hira* upstream arm (4209 bp) and 5′-GGCGCGCCGTGGTCATCTGGAATCTGCT-3′ and 5′-CGTACGATATTGGTTCCCGGTACCAG-3′ for the *Hira* downstream arm (3530 bp). These fragments were ligated in the pW25 vector [23] using the following restriction sites: Sac II and Acc65I for the upstream arm and AscI and BsiWI for the downstream arm. The final construct, named pW25-Hira, was verified by PCR and restriction analysis (unpublished data).

Six independent autosomal pW25-Hira transgenic lines were established in a *y w^67c^* background. Batches of 15–20 virgin *y w; P{70FLP}11 P{70I-SceI}2B, Sco/CyO* females were crossed with approximately 10 males from a given donor line in plastic vials. Vials containing 24-h egg collections from these crosses were heat shocked for 90 min at 37 °C in a water bath on days 3, 4, and 5 after egg laying. *pW25-Hira /P{70FLP}11 P{70I-SceI}2B, Sco* virgin F1 females with white or mosaic eyes were collected and crossed with *w ; P{70FLP}10* males. Non-mosaic, coloured-eyed progenies were then crossed again with *w ; P{70FLP}10* to establish individual lines. Each line with a *white* ^+^ chromosome resistant to constitutive Flipase activity was tested for its complementation with the *w Hira^ssm^* chromosome. Chromosomes that did not complement the maternal effect embryonic lethality associated with *Hira^ssm^* were selected, outcrossed with *w^1118^* for five generations, and balanced with the FM7c Chromosome.

### *pW25-Hira^HR1^-Flag* transgenes.

The *pW25-Hira^HR1^-Flag* transgene was constructed by replacing an AgeI-BsiWI restriction fragment in the 3' *Hira* arm from the *pW25-Hira* vector with a 729 bp fragment excised from the *pW8-Hira-Flag* transgene [17] to introduce the 3X-Flag tag at the 3' end of *Hira*. The final construct was verified by sequencing, and transgenic lines were established.

### RT-PCR.

Total RNA was extracted by the Trizol method (Invitrogen) and first-strand cDNAs were synthesized with the Superscript II reverse transcriptase (Invitrogen) and oligo-dT primers. The primer sequences used for PCR amplification of the cDNAs or genomic DNA are available on request.

### Antibodies for immunofluorescence.

Anti-Flag M2 mouse monoclonal antibody (F-3165, Sigma Aldrich) was used at 1:2000, rabbit anti-acetylated histone H4 polyclonal antibody (06–598, Upstate) at 1:500, rabbit anti-H3K4me3 polyclonal antibody (ab8580, Abcam) at 1:250, and mouse monoclonal anti-GFP antibody (Roche 1814460, clones 7.1 and 13.1) at 1:500 (IF). The anti-*Drosophila* ASF1 antibody [34] is a gift from F. Karch and was used at a 1:1000 dilution. The HIRA 830 anti-peptide antibody was described before and used at 1:500 [17].

For the production of the PG1 anti-HIRA polyclonal antibody, a plasmid *PW8-Hira-Flag* [17] was used as a template to amplify a 1943-pb fragment from 1241 to 3183 (amino acids 381–935) by PCR using primers 5′-ACATATGGTGAACGGTCTGGGAAAGTC-3′ and 5'TGGATCCGTACCCGTTGTCACAGCCAT-3′. The fragment was cloned into the NdeI and BamHI of pET15b vector (Novagen) in frame with the His•Tag® at the N-terminus end of the recombinant protein. The recombinant plasmid was transformed into Escherichia coli BL21-CodonPlus® (DE3)-RIL competent cells (Stratagene) and expression of the recombinant protein was induced by IPTG (isopropyl β-D-thiogalactoside) as described by the manufacturer and analysed by SDS-PAGE. Two rabbits were immunized with the purified HIRA-HIS-TAG protein purified on a Nickel column. Crude sera were purified on a Proteine-G column (Proteogenix) and were used at 1:1000.

### Immunofluorescence.

Eggs and embryos were collected, fixed in methanol, and immunostained as described [16]. For each experiment, we observed a minimum of 25 eggs/embryos at the desired stage. Testes and ovaries were dissected in PBS-Triton 0.1%, fixed in 4% paraformaldehyde for 20 min (testis) or 30 min (ovaries) at room temperature, rinsed in TBST (0.1% Triton), and stained as for embryos. DNA was stained either with propidium iodide as described [16] or with TO-PRO-3 (Molecular Probes) used at a 1:10,000 dilution. Preparations were observed under a Zeiss LSM Meta confocal microscope. Images were processed with the LSM and Photoshop (Adobe) softwares.

### Western blot.

WT and transgenic O/N embryos were collected, washed, dechorionated, and homogenized in Laemmli 2X sample buffer (125 mM Tris-HCl [pH 6.8], 2% SDS, 10% glycerol, 100mM DTE, 1% bromophenol blue) with an Eppendorf fitting pestle-homogenizer using the bio-vortexer^TM^ mixer (Roth). Protein samples were centrifuged 5 min at 5000 rpm, boiled for 10 min at 95 °C, and subjected to electrophoresis on an 10% SDS-PAGE gel. Immunoblotting was performed using a tank transfer system (Mini Trans-Blot Cell, Bio-Rad) and Hybond-C Extra nitrocellulose membranes (Amersham Biosciences) in transfer buffer (25 mM Tris, 20 mM glycine, 20% ethanol, 0.05% SDS). Antibodies incubation was in TBST (20 mM Tris-HCl [pH 7.5], 130 mM NaCl, 0.1% Tween 20) supplemented with 1% (w/v) nonfat dry milk as blocking agent. Detection was performed using the ECL western blotting detection system (Amersham Pharmacia). Anti-FLAG M2 (F-3165, Sigma Aldrich) and anti-α-tubulin Dm1A (T-9026, Sigma Aldrich) mouse monoclonal antibodies were used at a 1:20,000 dilution. Goat anti-mouse horseradish peroxidase–conjugated antibody (170-5047, Bio-Rad) was used at 1:15,000 dilution. Note that our HIRA antisera did not work on western blots using the extraction and detection procedures that worked very well with the HIRA-FLAG recombinant protein detected with the anti-FLAG antibody.

## Abbreviations

HR1: homologous recombination 1
RC: replication coupled
RI: replication independent
SCR: sperm chromatin remodelling
SNBP: sperm nuclear basic protein
*snky*: *sneaky*
*sra*: *sarah*
*ssm*: *sésame*
TC: trancription coupled
